# Frequency response areas in the inferior colliculus: nonlinearity and binaural interaction

**DOI:** 10.3389/fncir.2013.00090

**Published:** 2013-05-10

**Authors:** Jane J. Yu, Eric D. Young

**Affiliations:** Center for Hearing and Balance, Department of Biomedical Engineering, Johns Hopkins UniversityBaltimore, MD, USA

**Keywords:** inferior colliculus, tuning, nonlinearity, binaural, model, random spectral shape, level tolerant, dynamic range

## Abstract

The tuning, binaural properties, and encoding characteristics of neurons in the central nucleus of the inferior colliculus (CNIC) were investigated to shed light on nonlinearities in the responses of these neurons. Results were analyzed for three types of neurons (I, O, and V) in the CNIC of decerebrate cats. Rate responses to binaural stimuli were characterized using a 1st- plus 2nd-order spectral integration model. Parameters of the model were derived using broadband stimuli with random spectral shapes (RSS). This method revealed four characteristics of CNIC neurons: (1) Tuning curves derived from broadband stimuli have fixed (i. e., level tolerant) bandwidths across a 50–60 dB range of sound levels; (2) 1st-order contralateral weights (particularly for type I and O neurons) were usually larger in magnitude than corresponding ipsilateral weights; (3) contralateral weights were more important than ipsilateral weights when using the model to predict responses to untrained noise stimuli; and (4) 2nd-order weight functions demonstrate frequency selectivity different from that of 1st-order weight functions. Furthermore, while the inclusion of 2nd-order terms in the model usually improved response predictions related to untrained RSS stimuli, they had limited impact on predictions related to other forms of filtered broadband noise [e. g., virtual-space stimuli (VS)]. The accuracy of the predictions varied considerably by response type. Predictions were most accurate for I neurons, and less accurate for O and V neurons, except at the lowest stimulus levels. These differences in prediction performance support the idea that type I, O, and V neurons encode different aspects of the stimulus: while type I neurons are most capable of producing linear representations of spectral shape, type O and V neurons may encode spectral features or temporal stimulus properties in a manner not easily explained with the low-order model. Supported by NIH grant DC00115.

## Introduction

The central nucleus of the inferior colliculus (CNIC) is an important site of convergence in the auditory system (Adams, 1979; Brunso-Bechtold et al., 1981; Winer, 2005). Ascending inputs to the CNIC terminate in tonotopically organized layers, but afferents from different brainstem sources innervate overlapping domains within the layers (Oliver et al., 1997; Henkel et al., 2003; Cant and Benson, 2008; Malmierca et al., 2009; Loftus et al., 2010). Despite the diversity of inputs, the cellular organization of CNIC is relatively homogeneous, with only a small number of morphological cell types that are not gathered into subnuclei or into an organized microstructure (Oliver and Morest, 1984; Malmierca et al., 1993; Ito and Oliver, 2012; Wallace et al., 2012). Studies of neurons' responses and electrophysiological characteristics are consistent with this anatomical evidence, in that neurons showing a relatively small number of response patterns are scattered throughout CNIC (e.g., Ramachandran et al., 1999; Sivaramakrishnan and Oliver, 2001).

The lack of distinct morphological cell types and organized microstructure suggests that progress on the representation of sound in the CNIC will depend on physiologically defined neuron classes. One basis for such classes is the pattern of frequency selectivity of CNIC neurons in response to tones (Davis, 2005). Tone responses can serve to define response classes; however, the most commonly encountered stimuli in the natural environment are broadband. Because tone responses typically do not accurately predict selectivity for broadband or natural stimuli (Nelken et al., 1997; Holmstrom et al., 2007; May et al., 2008), it seems important to derive models of spectral integration on the basis of responses to broadband stimuli.

The tuning of neurons to broadband sounds can be studied using a method like reverse correlation (De Boer and De Jongh, 1978; Aertsen and Johannesma, 1981) that derives an equivalent filter consistent with the neuron's responses to broadband or natural stimuli. In the CNIC, neurons have been characterized using the so-called spectro-temporal receptive field (STRF) which is based on the assumption that neurons perform a 1st-order (linear) spectro-temporal weighting of the stimulus (e.g., Klein et al., 2000; Theunissen et al., 2000; Escabi and Read, 2005; Eggermont, 2011). However there are a number of important questions remaining. The STRF has been successful in describing various aspects of neural responses [e.g., stimulus-dependence of responses, (Theunissen et al., 2000); motion sensitivity, (Andoni and Pollak, 2011); spatial organization of response properties, (Chen et al., 2012); and comparison of response complexity between CNIC and auditory cortex, Atencio et al., 2012; etc.], but often STRFs do not predict the responses to test stimulus ensembles accurately (Machens et al., 2004; Versnel et al., 2009; Eggermont, 2011). Significant improvements in the models and in prediction have been made recently (Ahrens et al., 2008; Calabrese et al., 2011), but the reasons for the limited performance are not fully understood. In this work, we study three likely causes of limited prediction performance.

First, it seems likely that the performance of existing models is limited by the nonlinearity of auditory neural integration (Johnson, 1980; Christianson et al., 2008). Models have sometimes taken a “linear-nonlinear” form in which a 1st-order weighting of the stimulus is followed by a static nonlinearity to match the growth of response with stimulus level (Sharpee et al., 2004; Nagel and Doupe, 2006; Lesica and Grothe, 2008a). In other models, an input nonlinearity is postulated (most simply, using the log of the stimulus amplitude instead of the amplitude or power; Escabi et al., 2003; Ahrens et al., 2008), but nonlinearities intrinsic to the frequency response itself have not been considered.

Second, models have usually been studied at one or two sound levels only (but see Bandyopadhyay et al., 2007; Ahrens et al., 2008; Lesica and Grothe, 2008a; Pienkowski and Eggermont, 2011), while auditory nonlinearities often change significantly across sound levels (Nelken et al., 1997; Bandyopadhyay et al., 2007). For example, in the dorsal cochlear nucleus, neurons behave in a relatively linear manner at low sound levels, but exhibit nonlinearity at higher levels where so-called “type II” inhibitory interneurons are active. Thus there is a need for systematic examination of the effects of sound level on auditory spectral integration.

Finally, most studies of spectral representation in CNIC (and auditory cortex) have not investigated the separate contributions of the two ears in a systematic manner; stimulus presentations have been either monaural or free-field (but see Schnupp et al., 2001; Qiu et al., 2003). Thus, the possibility that nonlinear interactions occur between neural inputs from the two ears has also not been investigated sufficiently.

Here we describe spectral integration in three common response types of CNIC neurons (I, V, and O; Ramachandran et al., 1999) in unanesthetized (decerebrate) cats. The rate responses to broadband stimuli are used to construct weighting function models (Yu and Young, 2000; Young and Calhoun, 2005) that measure 1st and 2nd-order relationships between stimulus spectra and average discharge rates across a range of stimulus levels. Neural responses to the temporal aspects of the stimuli are not addressed in this paper. In the three response types, tuning observed in response to broadband stimuli is substantially more level tolerant than tuning to tones. Moreover, although contributions of the ipsilateral ear to neural responses are usually weak relative to contributions of the contralateral ear, they are still significant in many neurons. Finally, 2nd-order nonlinearities are significant and take a form that is poorly captured by the linear-nonlinear model. These results point the way for improvements in models of central auditory neurons. They also support previous suggestions (Davis, 2005) that the representation of stimuli in CNIC occurs in parallel pathways with characteristics that provide differing information about aspects of the stimulus.

## Materials and methods

### Animal preparation and recording

The experimental protocol was approved by the Johns Hopkins Animal Care and Use Committee. Data were obtained from eight adult male cats (3–4 kg) with clean ears and clear tympanic membranes. The cats were anesthetized with xylazine (2 mg im) plus ketamine (40 mg/kg im), then treated with dexamethasone (2–4 mg im) and atropine (0.1 mg im) to delay the onset of edema and minimize mucous secretions, respectively. A tracheostomy was performed to facilitate quiet breathing. Supplemental doses of ketamine (15 mg/kg iv) were given as needed during surgery. Body temperature was maintained between 37 and 40°C. A supracollicular decerebration was performed by aspiration. Anesthesia was then discontinued.

The ear canals were transected and hollow ear bars inserted to prepare for stimulus delivery. Polyethylene tubing (PE-200, approximately 40 cm in length) was inserted into each bulla to prevent static pressure buildup in the middle ear. A craniotomy was performed, tissue was aspirated, and dura was opened to expose the IC. In experiments that lasted several days, cats were given intravenous lactated ringers to maintain body fluids. Experiments were terminated when vascular pulsations or edema at the recording site prevented single neuron isolation. Animals were euthanized with a lethal intravenous injection of sodium pentobarbital.

**Figure 1:**
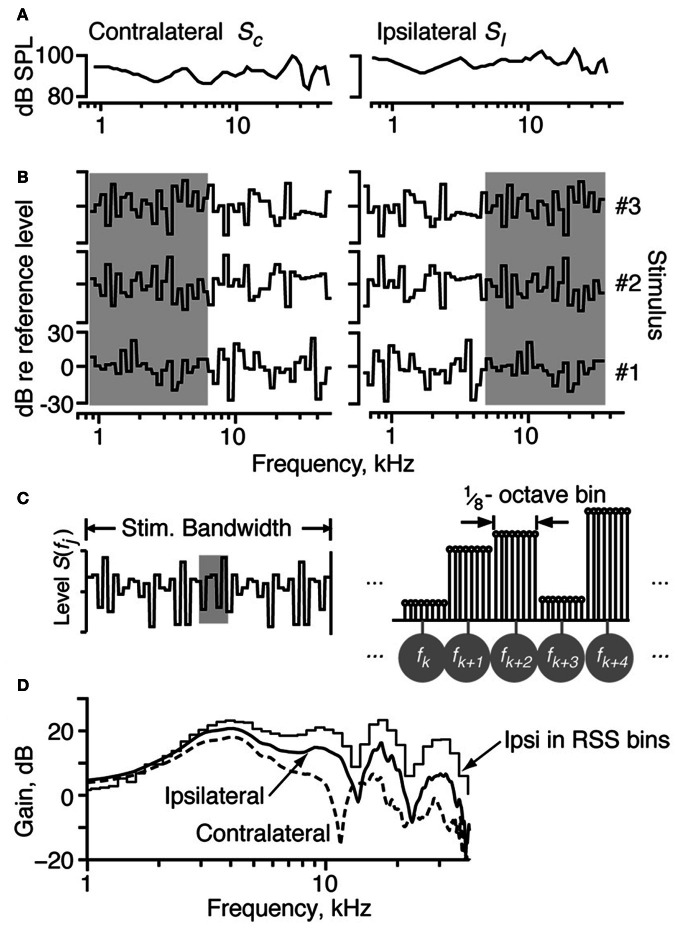
**The properties of the RSS stimuli. (A)** The acoustic calibration of the two ears in one animal, showing the sound pressure level as a function of frequency at 0 dB attenuation. These were determined using a calibrated probe tube placed within 2 mm of the eardrum. The spectra of sounds were modified by filtering with these transfer functions. **(B)** Spectra of three example RSS stimuli, out of the set of 200, shown as the dB spectrum level vs. log frequency. The sound levels of individual frequency bins (of width 1/8 octave) are symmetrically distributed around a mean value of 0 dB, with SD 12 dB. The reference 0-dB sound level is varied with an attenuator, usually over a 50–70 dB range. Note that the spectra presented to the ipsilateral ear are frequency-shifted versions of the spectra presented to the contralateral ear (e.g., compare gray areas). **(C)** Spectral shape of the RSS stimuli in more detail. Each frequency bin consists of 8 tones, logarithmically spaced at 1/64 octave. Individual tones are shown in the line spectrogram at right, which corresponds to the shaded region of the stimulus spectrum at left. The eight tones in each bin (1/8 octave wide) have equal sound level. Frequencies of the bin centers are indicated by the symbols in gray circles. **(D)** Spectra of white noise filtered with two cat HRTFs. The ipsilateral and contralateral HRTFs approximate the spectral shapes in the ipsilateral and contralateral ears of a broadband noise stimulus played at 15° azimuth, 30° elevation. The stepped-function (“Ipsi in RSS bins”) shows the stimulus energy for the ipsilateral spectrum in bins corresponding to the RSS stimuli at a sampling rate of 100 kHz.

An electrostatic speaker was coupled to each ear bar for stimulus delivery. The sound in each ear was calibrated near the tympanic membrane with a probe microphone. Sample acoustic calibrations are shown in Figure 1A.

A platinum-iridium microelectrode was inserted dorso-ventrally into the IC (at an angle of 5–15° from vertical) under visual guidance, and single neurons were isolated from the amplified, filtered electrode signal using a Schmitt trigger. Each neuron was characterized by its best frequency (BF) and threshold in response to tones in the contralateral ear. Here, we define BF as the frequency at which a response is observed at the lowest sound level of the stimulus. The excitatory (E) or inhibitory (I) nature of the responses to contralateral and ipsilateral stimuli was determined by presenting monaural tones to each ear at BF. All neurons were binaural, although the responses of some neurons to ipsilateral stimuli were weak. Most neurons were EE or EI; that is, they were either excited by tones in both ears (EE), or excited by tones in the contralateral ear and inhibited by tones in the ipsilateral ear (EI).

**Figure 2:**
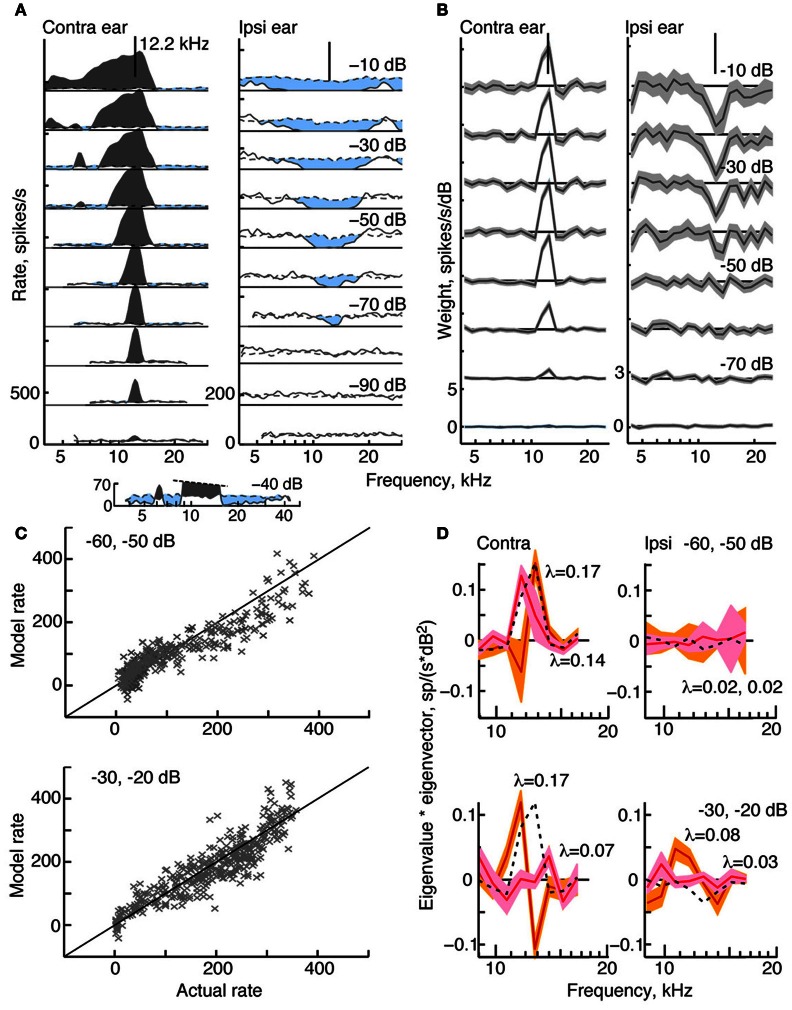
**Response characteristics for a type I neuron, BF 12.2 kHz. (A)** Tone response maps showing rate responses to 200-ms tone bursts, plotted against the tone frequency at a fixed attenuation. Rates were computed from single tone presentations and smoothed (5-bin triangular filter) for display. Attenuations are shown at right. Zero dB attenuation was 94 dB SPL at the neuron's BF. Response maps were created by presenting tones in the contralateral ear only (left) and ipsilateral ear only (right). Discharge rates across the duration of the tone are shown by solid lines. Spontaneous rates are shown by dashed lines. The horizontal solid straight line indicates 0 spikes/s, and the rate scale is given at bottom left (different in the two ears). Excitatory responses are colored gray and inhibitory responses are light blue. The vertical lines at the top of the plots show the BF in the contralateral ear (12.2 kHz). **(B)** Weight functions estimated from responses to binaural RSS stimuli presented across a range of attenuations. First-order weights (*w*_*C*_ and *w*_*I*_ in Equation 1) are plotted on the same frequency axis as in **(A)**. Weight estimates are shown as black lines, and gray regions indicate ±1 SEM. Contralateral and ipsilateral weight functions were derived from the same 200 responses to the binaural stimulus set. Note that the weight scales on the ordinate differ. **(C)** Rates predicted by the model (ordinate) vs. experimental rates (abscissa) in leave-one-out model testing. For each plot, data for two attenuations (indicated in the legend) were combined for the leave-one-out procedure. **(D)** Second-order effective filters (i. e., eigenvectors of **M**_*C*_ and **M**_*I*_ in Equation 3) for the same two fits shown in **(C)**. Eigenvectors multiplied by their corresponding eigenvalue are plotted against frequency. The 1st-order weights, scaled to the same maximum value in each plot, are shown as black dotted lines. Only eigenvectors with the two largest positive eigenvalues λ are shown (largest λ, orange; second-largest λ, pink). The colored regions indicate ±1 SEM. The negative eigenvalues are smaller than the positive eigenvalues (<0.03, top case; <0.1, bottom case) and the corresponding eigenvectors are noisy (not shown).

**Figure 3:**
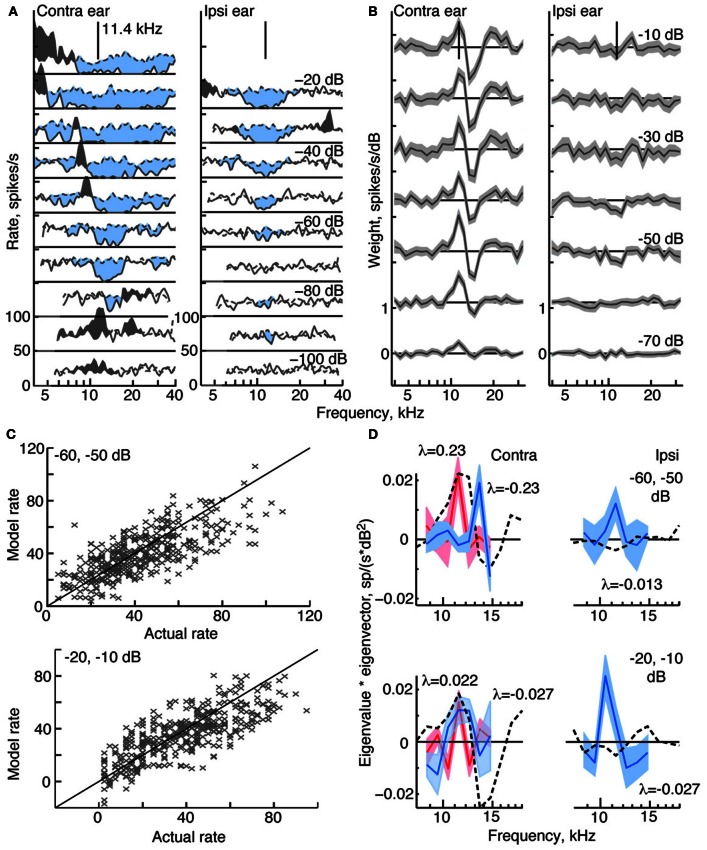
**Response characteristics for a type O neuron, BF 11.4 kHz. (A,B)** Tone response maps and RSS weight functions, plotted as in Figure 2. Note the large inhibitory area centered on BF in the contralateral and ipsilateral tone response maps. Also note the difference in shape of the tone and weight-function maps. 0 dB attenuation is 98 dB SPL at the BF of the neuron. **(C)** Prediction performance as in Figure 2. *fv*-values are 0.54 for the stimuli at −60/−50 dB, and 0.51 at −20/−10 dB. **(D)** 2nd-order weight functions with the largest eigenvalues, given in the legends. Weights with positive eigenvalues are shown in red, and weights with negative eigenvalues are shown in blue.

**Figure 4:**
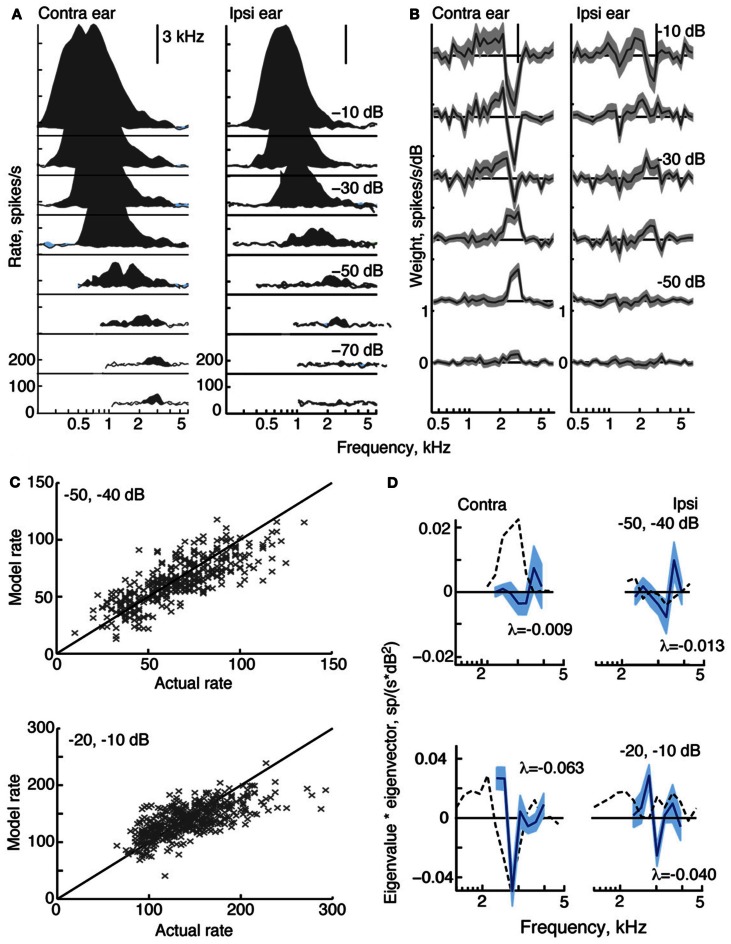
**Response characteristics for a type V neuron, *BF* = 3 kHz. (A)** Response maps, plotted as in Figure 2. Zero dB attenuation was 98 (contra) and 95 (ipsi) dB SPL at the BF of the neuron. **(B)** At high sound levels, 1st-order weight functions suggest a substantial inhibitory area around BF–a pattern consistent with the weak responses to tones near BF in **(A)**. **(C)** Prediction performance as in Figure 2. *fv*-values are 0.61 for the stimuli at −50/−40 dB, and 0.41 at −20/−10 dB. **(D)** 2nd-order weights with the largest negative eigenvalues, given in the legends.

Contralateral and ipsilateral response maps (shown below in Figures 2–4) were obtained by recording discharge rates in response to sequences of tones in the contralateral and ipsilateral ears, respectively. The frequencies of the tones in each ear were logarithmically spaced and spanned up to 4 octaves. Tone sequences were presented monaurally as 200-ms tone bursts, once per second, at fixed attenuation levels. Driven rates were averaged across the tone duration, and spontaneous rates were averaged across the last 400 ms of the stimulus-off period. Response maps were based on one presentation of each tone frequency at each sound level.

For the estimation and testing of weight-function models, broadband stimuli with random spectral shape (RSS) were presented to both ears simultaneously in 400-ms bursts, once per second, at fixed attenuation levels. Discharge rates were averaged across the 400-ms stimulus-on interval, and spontaneous rates were averaged across the last 400 ms of the stimulus-off intervals. Models were usually constructed from a single presentation of an ensemble of 200 RSS stimuli. In the few cases where multiple presentations of the same stimulus ensemble were performed, the resulting models were essentially the same as those based on a single presentation.

### Response types

CNIC neurons have been classified according to tone-based response maps using various criteria [e.g., Yang et al., 1992; Egorova et al., 2001; Lebeau et al., 2001; reviewed by Davis (2005)]. Here, we group neurons based on response map patterns and binaural properties—a classification scheme found most appropriate for the CNIC in decerebrate cats (Davis et al., 1999; Ramachandran et al., 1999). Type I (“eye”) neurons demonstrate sharply tuned excitatory responses to contralateral tones at frequencies surrounding BF, and inhibitory responses to ipsilateral tones at frequencies equal to and surrounding the contralateral BF (EI). This pattern remains stable across all sound levels tested (see Figure 2). By contrast, Type V (“vee”) neurons, which usually have a BF less than 3–4 kHz (in cats), demonstrate broad excitatory responses to contralateral and ipsilateral tones (EE). Type V response maps frequently also suggest that some degree of inhibition exists at and above BF at high sound levels (see Figure 4). Finally, Type O neurons are strongly nonmonotonic (Figure 3): tones near BF presented to the contralateral ear produce excitatory responses at low sound levels and inhibitory responses at higher sound levels. For Type O neurons, responses to ipsilateral-ear stimuli are inhibitory at all sound levels tested. The CNIC also contains relatively few onset neurons (<10%), which do not give sustained responses to tones. Note that these classification criteria are qualitative: they are determined mainly by the binaural ipsilateral/contralateral response type (EI or EE) and the degree to which contralateral tones inhibit responses around BF. A number of quantitative properties for these different response types have been documented (Davis et al., 1999; Ramachandran et al., 1999), but these were not necessary for neuron classification.

### Random-spectral-shape (RSS) and virtual-space (VS) stimuli

Random-spectral-shape stimuli spanning a frequency range of 5.7 octaves (0.8–43 kHz) were used to explore the spectral sensitivity of IC neurons to broadband stimuli. The spectra of these stimuli are comprised of 1/8-octave bins, where each bin contains 8 tones of the same level spaced 1/64 octave apart. Sound levels across the bins were independently drawn from a Gaussian distribution with a mean of zero dB and a standard deviation of 12 dB. Here, zero dB serves as the reference sound level of the stimulus, which usually varied over a 50–70 dB range of absolute intensities. Stimulus waveforms were constructed by summing the tones with a baseline sampling rate of 100 kHz; the starting phases for the tones were randomized to prevent a click at time 0. Because the stimuli are defined on a log frequency axis, changing the sampling rate simply shifts the stimuli along the log frequency axis without changing their spectral shapes, binwidths, or the log-frequency spacing of the tones. As such, whenever possible, the sampling rates of the RSS stimuli were changed to place the BF of the neuron between one-half and two-thirds of the total frequency range of the stimulus.

In order to estimate parameters for the weight-function model, we constructed 192 binaural RSS stimuli with unique spectral shapes. The spectra of three such stimuli are shown in Figures 1B,C. Each RSS stimulus is characterized by *S*(*f*), the sound levels in dB of the bins centered at frequencies *f*. Binaural stimuli are represented as two vectors ${\vec{s}}_{C}$ and ${\vec{s}}_{I}$ whose elements are dB sound levels *S*_*C*_(*f*_*j*_) and *S*_*I*_(*f*_*j*_) at each frequency in the contralateral and ipsilateral stimuli, respectively. Note that for each binaural stimulus pair, the low-frequency half of the stimulus in one ear is equal to the high-frequency half of the stimulus in the other ear (gray boxes in Figure 1B). This circular frequency shift ensures that ipsilateral spectra are orthogonal to contralateral spectra across half the stimulus bandwidth. Because the half-bandwidth of the stimuli (2.85 octaves) was wider than the bandwidth of the neurons, this symmetry did not confound characterization of a neuron's response patterns.

In addition to the 192 stimuli with RSS, we constructed eight binaural stimuli in which all levels are at the reference level: that is, *S*(*f*) = 0 dB for all *f*. These flat spectra stimuli were used to check the estimation of parameter *R*_0_ in the weight-function model described below.

Virtual-space stimuli (VS) were generated by filtering a broadband (50-kHz wide) flat-spectrum noise stimulus with head-related transfer functions (HRTFs) that were measured in cat [Figure 1D from Rice et al. (1992)]. HRTF filtering produces noise at the eardrum similar to that which would have been produced if the noise were presented in free field. HRTFs representing 100 spatial locations (range: −60 to +60° azimuth, −30 to +45° elevation) were presented to the ears in binaural pairs that provided a virtual space simulation (assuming that the contralateral and ipsilateral ears of the cat are identical). For example, if the sound presented to the contralateral ear were filtered by the HRTF for −7.5° elevation and 15° azimuth, then the sound presented to the ipsilateral ear was filtered by the HRTF for −7.5° elevation and −15° azimuth. Because the same 50-kHz noise stimulus was applied to all HRTF filters, the resulting binaural signals also had appropriate interaural time, interaural level, and spectral cues. The responses to VS stimuli were used to test the models derived from responses to RSS stimuli.

### Weight-function model

The spectral sensitivity of neurons responding to broadband stimuli was characterized using the following model.

(1) $$\begin{array}{l} r=R_{0}\text{​}+\text{​}\sum_{j=n_{1}}^{n_{2}}w_{Cj}S_{C}(f_{j})\text{​}+\text{​}\sum_{j=n_{1}}^{n_{2}}w_{Ij}S_{I}(f_{j})\text{​}+\text{​}\sum_{j=n_{3}}^{n_{4}}\sum_{k=j}^{n_{4}}m_{Cjk}S_{C}(f_{j})S_{C}(f_{k})\\ \;+\sum_{j=n_{3}}^{n_{4}}\sum_{k=j}^{n_{4}}m_{Ijk}S_{I}(f_{j})S_{I}(f_{k})+\sum_{j=n_{5}}^{n_{6}}\sum_{k=n_{5}}^{n_{6}}b_{jk}S_{C}(f_{j})S_{I}(f_{k}) \end{array}$$

This model has previously been applied to auditory nerve fibers (ANF) (Young and Calhoun, 2005), brainstem neurons (Yu and Young, 2000; Tollin and Koka, 2010), and neurons in the auditory cortex (Barbour and Wang, 2003); however, in the present study, binaural processing is also explicitly represented. In this model, the discharge rate of the neuron *r* is the sum of six terms: constant *R*_0_ which is the response to the flat-spectrum stimuli with all levels at 0 dB; 1st-order weightings of the spectral levels in each ear (the second and third terms); 2nd-order weightings of the levels in each ear, (the fourth and fifth terms); and a 2nd-order contribution related to a binaural interaction (the sixth term). Parameters *w*_*Cj*_ and *w*_*Ij*_ are 1st-order weights on stimulus levels at frequency *f*_*j*_ in the contralateral and ipsilateral ears, respectively. First-order weights represent gains of the rate response to energy at each frequency in spikes/(s·dB), and first-order weight functions show these gains for each stimulus frequency bin. Parameters *m*_*Cjk*_ and *m*_*Ijk*_ are 2nd-order weights which describe an interaction between spectral level pairs associated with the same ear [e.g., *m*_*Cjk*_ is the weighting on the multiplicative interaction *S*_*C*_(*f*_*j*_) *S*_*C*_(*f*_*k*_)]. Finally, parameter *b*_*jk*_ is a binaural weight measuring an interaction of spectral level pairs from opposite ears [i. e., *b*_*jk*_ is the interaction of *S*_*C*_(*f*_*j*_) in the contralateral ear and *S*_*I*_(*f*_*k*_) in the ipsilateral ear].

The model of Equation 1 is a linear function of the unknown parameters *R*_0_, {*w*_*Cj*_}, {*w*_*Ij*_}, {*m*_*Cjk*_}, {*m*_*Ijk*_}, and {*b*_*jk*_}. The parameters are estimated from discharge rates *r*, which are the responses to 192 RSS and 8 flat-spectrum 0-dB stimuli. At each reference sound level (expressed in dB attenuation), weights were estimated by solving 200 simultaneous linear equations in the form of Equation 1—that is, one equation for each stimulus presented. Weight estimates were obtained by using either the method of normal equations or singular-value decomposition (Press et al., 2007, chapter 15)—both of which minimize the mean square error between the actual discharge rates and those predicted by the model. For the estimates computed here, the two methods give identical results. Plots of the estimated weights against frequency at each of the reference sound levels were aggregated to form weight-function maps.

Weights were also computed by combining responses at two or more reference sound levels—a calculation which mainly affected estimates for 2nd-order weights. A model with combined sound levels usually predicted rate responses to RSS stimuli (but not HRTF stimuli) more accurately than models estimated at only one sound level: median improvements in the goodness of fit for the predictions (i. e., *fv*; defined in the next section) are 0.12, 0.15, and 0.12 for I, V, and O neurons, respectively. The performance improvement presumably reflects the fact that a larger amount of data (400 vs. 200 data points) and a wider range of stimulus levels were used to fit the model. Response predictions generated by combining data from multiple levels are mixed with single-attenuation fits in Figures 2C, 3C, 4C, 7, 9.

The frequencies that are assumed to contribute to a neuron's responses are additional parameters in the model. These parameters, which are shown as limits on the summations {*n*_1_, *n*_2_, *n*_3_, *n*_4_, *n*_5_, *n*_6_} in Equation 1, define the sequential set of weights (including BF) that lead to the model's best prediction performance. These frequency limits are determined by systematically varying the span of frequencies used during weight estimation and prediction. Parameters {*n*_1_, *n*_2_} are varied first, followed by {*n*_3_, *n*_4_} and finally {*n*_5_, *n*_6_}. Specifically, beginning with one weight at BF, weights are added below and above BF, one at a time, only as long as they improve prediction performance. When the 2nd-order weight limits are being determined, the 1st-order weight limits are fixed at the best performance of the 1st-order model. Similarly, when the binaural weight limits are being determined, both the 1st- and 2nd-order weight limits are fixed. Note that all weights in the current set, as specified by the {*n*_*j*_}, are estimated simultaneously at each step, so that as 2nd-order weights are added, the 1st-order weights usually change. Neither the weights nor the prediction error are strong functions of {*n*_*j*_}, especially near the edge of the response area, so the order in which the {*n*_*j*_} are tested does not change the model or error.

### Prediction

Increasing the limits on summation in the model amounts to increasing the number of weights that need to be estimated. To prevent overfitting the model, we consider only weights in the model that are needed to accurately predict responses to broadband stimuli. To test the model, we use a leave-one-out cross-validation procedure where each of the 200 rates is set aside as a test response while the remaining 199 rates are used to estimate weights. The procedure is repeated for each of the 200 rates. This method produces results similar to bootstrap; however, it is preferred over bootstrap because it yields 200 separate prediction tests while also using the largest number of data points to fit the model. The SEMs of the weights are estimated from the leave-one-out calculations as $\left(n-1\right)\sigma_{p}/\sqrt{n}$, where *n* is the number of data points (200) and σ_*p*_ is the standard deviation of the weights in the 200 leave-1-out trials [Equation 10.9 in Efron and Tibshirani (1986)].

The quality of prediction is measured with fraction of variance, defined as:

(2) $$fv=1-\frac{\sum {\left(r_{j}-r_{mj}\right)}^{2}}{\sum {\left(r_{j}-\hat{r}\right)}^{2}}$$

where *r*_*j*_ are the rates in the test data set, *r*_*mj*_ are the rates predicted by the model, and $\hat{r}$ is the mean of the *r*_*j*_. The value of *fv* is 1 for a perfect fit and decreases as the fit worsens. The *fv* is zero if the model predicts the data no better than the mean rate, and the *fv* can be negative for very bad fits. We have chosen *fv* as a measure of fit because, unlike the often-used correlation coefficient (*R*), *fv* is sensitive to constant rate errors (i. e., differences in the mean rates between model and data). Such errors can be a problem for a model with even-order terms. Empirical comparisons of *fv* and *R*^2^ based on the present data and auditory nerve data (Young and Calhoun, 2005) show that *R*^2^ ≥ *fv* in all cases, and that the two measures are approximately equal for good fits (taken to be values of *fv* above ~0.5). Noise in the rate measurements cannot be fit by the model and therefore decreases *fv*. A Poisson assumption for the rate statistics suggests that the maximum possible *fv* is about 0.8—a value consistent with the data (Figure 7B, assuming values above 0.8 are random scatter).

During this fitting process, *fv* for the 1st-order model usually did not change dramatically as the number of weights (*n*_1_ and *n*_2_) changed. However, the addition of 2nd-order weights to the best-performing 1st-order model (i. e., the 1st-order model with the largest *fv*) usually yielded increases in *fv* by up to 0.4, even when only one 2nd-order weight was introduced. The inclusion of additional 2nd-order weights (beyond the first one) also produced only small incremental changes in *fv.* This behavior, which was observed in 314/337 cases, suggests that the 2nd-order model provides information about spectral integration in these neurons that is not present in the 1st-order model. Note that binaural weights usually added little to the prediction quality of the best 1st- or 2nd-order models—improving *fv* by less than 0.01 in 225/310 cases.

Weight function models derived from RSS responses were used to predict responses to HRTF stimuli. Because the HRTF stimuli did not have an all-0-dB reference for the estimation of *R*_0_, a modified prediction procedure was used. For these predictions, the reference stimulus was chosen to be the average of the four HRTF stimuli with the flattest spectral shape within 0.5 octave of the neuron's BF. *R*_0_ was set to the average of the rates produced by those stimuli. HRTF responses were then predicted as differences between the response to the averaged reference stimulus and responses to each of the other 96 HRTF stimuli. This method produced usable predictions, but there were still errors in the rate predictions; specifically, there was often a shift in the means of the actual and predicted rates (see Figure 8B). As such, *R*^2^ (i.e., the square of the Pearson product-moment correlation coefficient), and not *fv*, was used to quantify errors between the actual and predicted rates (in Figure 8C only). Unlike *fv, R*^2^ is sensitive to correlated rate fluctuations but not to errors in average rate or overall rate gain.

### Second-order filters

The frequency selectivity implicit in the 2nd-order weights may be better understood by interpreting the terms in Equation 1 as filters (e.g., Lewis et al., 2002; Reiss et al., 2007). Equation 1 can be written in vector-matrix form as:

(3) $$r=R_{0}+{\vec{w}}_{C}^{T}{\vec{s}}_{C}+{\vec{w}}_{I}^{T}{\vec{s}}_{I}+{\vec{s}}_{C}^{T}M_{C}{\vec{s}}_{C}+{\vec{s}}_{I}^{T}M_{I}{\vec{s}}_{I}+{\vec{s}}_{C}^{T}M_{B}{\vec{s}}_{I}$$

where the stimulus vectors ${\vec{s}}_{C}$ and ${\vec{s}}_{I}$ are defined above. Weight vectors ${\vec{w}}_{C}$ and ${\vec{w}}_{I}$ contain the 1st-order weights. Matrices **M**_*C*_, **M**_*I*_, and **M**_*B*_ contain 2nd-order and binaural weights corresponding to frequency pairs. Because the 2nd-order matrices **M**_*C*_ and **M**_*I*_ are real and symmetric, they can be written as

(4) $$M=\sum_{j=1}^{N}\lambda_{j}{\vec{e}}_{j}{\vec{e}}_{j}^{T}$$

where λ_*j*_ are real eigenvalues of **M** and ${\vec{e}}_{j}$ are orthonormal eigenvectors. As such, each 2nd-order term in Equations 1 and 3 can be written as:

(5) $${\vec{s}}^{T}M\vec{s}={\vec{s}}^{T}\left(\sum_{j=1}^{N}\lambda_{j}{\vec{e}}_{j}{\vec{e}}_{j}^{T}\right)\vec{s}=\sum_{j=1}^{N}\lambda_{j}{\left({\vec{s}}^{T}{\vec{e}}_{j}\right)}^{2}.$$

Examination of Equation 5 shows that an eigenvector ${\vec{e}}_{j}$ can be viewed as an “equivalent 2nd-order filter.” However, because the filtering operation ${\vec{s}}^{T}{\vec{e}}_{j}$ is squared, the excitatory or inhibitory nature of the 2nd-order filter is determined by the sign of the eigenvalue, where a positive eigenvalue indicates an excitatory contribution and a negative eigenvalue indicates an inhibitory contribution. Furthermore, the magnitude of the eigenvalue quantifies the importance of the associated filter. In this paper, filters are shown for only the largest one or two eigenvalues.

## Results

Neurons in the CNIC show a variety of patterns of frequency sensitivity, as measured by responses to tones [reviewed by Davis (2005)]. Here, we use response types described by Ramachandran and colleagues in decerebrate cat (Davis et al., 1999; Ramachandran et al., 1999), as detailed in Materials and Methods. As stated previously, there are three types of tonic responses: types V, I, and O. Figures 2 through 4 provide examples of tone and broadband tuning for these neuron types. Data presented in this paper are from neurons for which complete tone and RSS responses were obtained across a range of sound levels. These include 12 V neurons (BFs 0.8–4.1 kHz), 13 I neurons (BFs 1.8–21 kHz), and 10 O neurons (BFs 2.2–21 kHz). Onset neurons were also encountered during the study—comprising 9% of the total sample—and all gave reliable tonic responses to RSS stimuli. However, onset neurons were not studied completely and are not discussed here.

### Response maps

#### Type I tuning

Figure 2A shows a tone response map typical of a type I neuron. Contralateral response maps (left) show a narrow excitatory area centered on BF (in this case, 12.2 kHz) and inhibitory sidebands at frequencies below and above the excitatory area (light blue). To better visualize the inhibitory areas, the response at −40 dB is shown on an expanded scale at the bottom of Figure 2A. Ipsilateral response maps (right), by contrast, show mainly inhibitory areas that are well tuned and centered on approximately the same BF. Like all type I neurons that were analyzed in the study, this neuron is EI in its responses near BF.

The effective tuning of a type I neuron for broadband noise can be inferred from the 1st-order weight functions (Figure 2B). First-order weight functions computed for the contralateral ear (at left) resemble contralateral response maps in that they show large positive peaks at BF. Similarly, 1st-order weight functions for the ipsilateral ear are negative at frequencies where responses to ipsilateral tones are inhibitory. This pattern of excitation and inhibition inferred by the weight-function maps is typical of all type I neurons studied.

To better understand the nature of 2nd-order weight functions, eight 2nd-order filters (Equation 5) were computed for the type I neuron featured in Figure 2. At low sound levels, negative eigenvalues for this neuron are small compared to the positive eigenvalues—a finding that supports a predominantly excitatory contribution of 2nd-order weights. However, inhibitory 2nd order effects are present at higher sound levels, where negative and positive eigenvalues reach comparable values (<0.1, not shown). The two filters corresponding to the largest positive eigenvalues are shown in Figure 2D. As indicated by the shaded areas (±1 SEM), most of the second-order weights are significantly different from zero. First-order weight functions at the same sound levels are superimposed for comparison (black dashed lines). Some of the contralateral 2nd-order filters are shown to have plus-minus shapes that differ significantly from 1st-order weight functions. For example, peaks are present at frequencies below BF rather than on BF, and substantial gain slopes occur near the BF. These differences suggest that 2nd-order filters change the frequency selectivity of the neuron.

The meaningfulness of the second-order weight function was evaluated by testing how well the model predicted responses to RSS stimuli not used in parameter estimation. Predictions were performed at the same sound levels featured in Figure 2B, and the results for two sound levels are shown (Figure 2C). Here, each data point is the predicted rate response to each left-out test stimulus (ordinate), plotted against the actual rate response to that stimulus. The *fv*-values (Equation 2), which measure the accuracy of the prediction, are 0.85 and 0.79 at the sound levels shown. For this type I neuron, the contribution of the 2nd-order terms in the weight-function model is significant: when all 2nd-order weights are excluded from the model, *fv*-values decrease to 0.32 and 0.58, respectively.

#### Type O tuning

Response maps and weight functions of a type O neuron are shown in Figures 3A,B, respectively. At low sound levels, the contralateral response map for the type O neuron (Figure 3A, left) shows an excitatory response to low-level tones near BF (11.4 kHz at −90 dB); at higher levels, the response map demonstrates a strong inhibitory response around BF, and a mixture of excitatory and inhibitory responses at frequencies away from BF. Given that the responses to ipsilateral tones (Figure 3A, right) were predominantly inhibitory, the neuron was classified as EI, as were all type O neurons studied. Unlike the type I neuron (Figure 2), contralateral weight functions computed from responses to RSS stimuli (Figure 3B, left) differ significantly from the tone-based response map: rather than having large areas of inhibition at BF, the weight functions show a combination of positive gains near BF and negative gains above BF across a range of sound levels. Ipsilateral weight functions (Figure 3B, right), on the other hand, are similar to the response maps in that they mainly show weak inhibition near BF at most sound levels.

The equivalent 2nd-order filters show substantial inhibitory and excitatory contributions (Figure 3D), possessing both large negative (blue filters), and large positive eigenvalues (red filters). The contralateral and ipsilateral 2nd-order filters are statistically different from zero, and their shapes differ significantly from their corresponding 1st-order weight functions.

Compared to the weight-function model for the type I neuron, the model for the type O neuron is a less accurate predictor of responses to novel stimuli (Figure 3C). For this type O example, the general trend in the data is captured by the model; however, the *fv*-values are only 0.54 and 0.51 at the two sound levels shown. The 2nd-order terms are important to the model, as exclusion of these terms produces *fv*-values (for the best-fitting 1st-order model) of only 0.38 and 0.28, respectively.

#### Type V tuning

Like all type V neurons in this study, the neuron in Figure 4 is EE, exhibiting broad excitatory responses to contralateral and ipsilateral tones (Figure 4A). At high sound levels, the weak response near BF, in addition to apparent limits on the spread of excitation to frequencies above BF, suggests that the response of the neuron is sculpted by inhibition. Although this behavior is observed in many V neurons (Davis et al., 1999), responses to tones that are strictly inhibitory (i. e., with rates below spontaneous rate) are small and rarely observed. Note that type V neurons were also encountered for which either, or both, response maps did not show signs of inhibition (not shown).

At low sound levels, weight functions for the type V neuron in Figure 4B resemble tone response maps in that weights are significantly positive at BF. Weight functions at higher sound levels show negative weights around BF in a manner consistent with the inhibitory sculpting described above. Second-order filters (Figure 4D) corroborate the inhibitory nature of this neuron's response: the largest eigenvalues are negative, and increases in sound level produce even larger negative eigenvalues.

In Figure 4C, the weight-function model predicts responses of the type V neuron with moderate accuracy. The *fv*-values for the 2nd-order model are 0.61 and 0.41 at the two sound levels shown. Exclusion of 2nd-order weights from the model reduces *fv*-values to 0.54 and 0.26, respectively.

### Level tolerance

**Figure 5:**
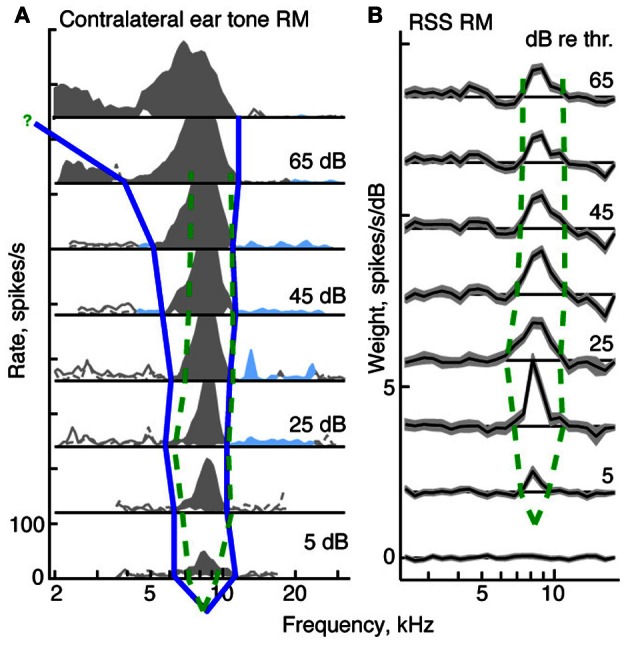
**Derivation of tuning curves from response maps for a type I neuron (*BF* = 8.3 kHz). (A)** Tone response map for the contralateral ear. The blue curve shows the tuning curve edges for the tone response map, derived as described in the text. The green dashed curve in both **(A)** and **(B)** shows the edges of the tuning curve for the weight-function map. **(B)** Weight-function map for the same neuron. The dB scale in both parts is dB re threshold for BF tones **(A)** or RSS stimuli **(B)**. The tuning curve near the BF tip is not well specified by the response maps. For the tone response maps, the threshold at BF is determined from a rate-level function; for the weight-function maps, the threshold is set halfway between levels that do and do not produce weights that are significantly different than zero.

In this section, we compare the tuning of neurons responding to tones against the tuning implicit in responses to RSS stimuli. Specifically we compare areas of activity observed in tone response maps with those seen in weight-function maps at equivalent sound levels (dB re threshold). For type I neurons, this comparison is robust and straightforward. In the tone maps of type I neurons, edges of activity are taken to be the frequencies at which rate responses first go to zero on either side of BF (Figure 5A, blue line). These edges define a central excitatory region flanked by inhibitory sidebands. Analogously, in weight-function maps, edges of activity are taken to be the frequencies at which 1st-order weights are first indistinguishable from zero on either side of BF (i.e., within ±1-SEM of zero; Figure 5B, dashed green lines). In this example, as sound level increases, tone-based tuning (blue curve) broadens, as is commonly observed for auditory neurons in many parts of the brain. By contrast, the tuning apparent in noise-based weight-function maps is level tolerant: that is, bandwidths are relatively fixed over the range of sound levels studied (dashed green curve).

**Figure 6:**
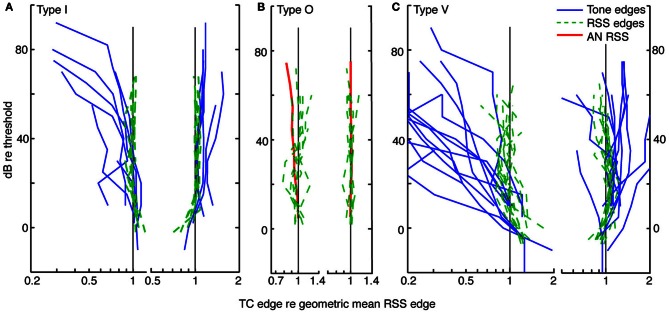
**Level tolerance of the edges of tone and weight-function response maps for populations of type I (A), O (B), and V (C) neurons.** Frequency edges computed as in Figure 5 are shown for *contralateral* stimuli only. Lower-(left) and upper-(right) frequency edges derived from tone response maps (blue lines) are overlaid on those derived from weight-function maps (dashed green lines) at equivalent sound levels, in dB re threshold. The point at BF is not included. For each neuron, lower (or upper) edge frequencies are plotted relative to the geometric mean across levels of the lower (or upper) edge frequencies of the weight-function maps. Slopes of lower frequency edges of tone maps differ significantly from those observed in weight-function maps (type I: *P* = 0.1; type V: *P* < 0.01; signed rank sum comparisons of slopes of best-fit lines). Upper-frequency edges do not differ. Tone maps were not analyzed for type O neurons (see text). The red curves in **(B)** show mean weight-function edges for auditory nerve fibers [ANF; data from Figure 5 of Young and Calhoun (2005)]. Here, frequencies are normalized by the average frequency edge at the lowest two sound levels. As sound level increases, the slopes of lower-frequency edges of CNIC weight-functions differ from those observed in ANF. Specifically, slopes suggest a relative narrowing in type I and type O data (*P* = 0.02), and relative widening in type V neurons (*P* = 0.07). For all weight-function types, upper frequency edges are not significantly different from those seen in ANFs. All Ps are Bonferroni corrected.

The comparison of tone- and noise-based tuning in Figure 5 is typical of type I neurons. This is shown in Figure 6A where upper and lower edges of tuning curves are shown for type I neurons. Frequency edges derived from weight-function maps (green dashed curves, as in Figure 5) are overlaid on those derived from tone-based response maps (blue lines, as in Figure 5A). To enable comparison of tuning curve slopes across neurons with different BFs, lower (or upper) frequency edges for each tuning curve (i. e., tone response maps and weight-function maps) are plotted relative to the geometric mean of lower (or upper) edge frequencies across all sound levels of the weight-function map. The lower and upper edges are plotted separately and the point at BF is not included.

Type I tuning in weighting functions is level tolerant: as sound level increases, upper and lower frequency edges remain relatively fixed. Level tolerance, however, was not observed in tone response maps, where increases in sound level typically yielded a broadening of response maps at the lower frequency edge: the slopes differ significantly between tone and weight-function maps on the low-frequency side (*P* = 0.1, Bonferroni corrected signed-rank-sum comparisons of slopes of best-fit lines), but not the high-frequency side.

For type O neurons, a direct comparison of tone- and noise-based tuning was not done because the patterns of excitation and inhibition in tone maps are complex and vary significantly from sound level to sound level (as in Figure 3A); thus, it is not clear how a meaningful and consistent bandwidth measure would be chosen. However, weight-function maps could be analyzed as described above for type I neurons. Because type O neurons often have distinct excitatory *and* inhibitory regions that are prominent near BF (as in Figure 3B, 3/7 cases), frequency edges in type O maps were chosen to include all statistically significant features that persisted across sound level (i. e., weights exceeding ±1 SEM). For the map in Figure 3B, the persistent feature that was selected consists of both the excitatory lobe near BF and the inhibitory lobe above BF. The choice of persistent feature is not critical, for as long as the same feature definition is applied consistently across sound level, the result does not change for the sample of type O neurons studied. In Figure 6B, upper and lower frequency edges are shown for a population of type O neurons. Note that the frequency edges are roughly constant at the different sound levels, thus implying that broadband frequency selectivity in type O neurons is tolerant to increases in noise level.

A similar analysis for a population of type V neurons is shown in Figure 6C. As with type I neurons, edges of activity in tone-based response maps were taken to be frequencies at which rate responses first go to zero on either side of BF. This area of activity included frequencies at and above BF where responses were excitatory but appear dampened by inhibition (e. g., Figure 4A). As with type O neurons, frequency edges for weight-function maps were defined to encompass distinct statistically significant features. However, unlike type O neurons, the excitatory and inhibitory nature of those features often changed with changes in sound level, as in Figure 4B. In such cases, all features near BF were included as part of a response area if they persisted over at least two levels. Because of the variability in weight-function maps, there is considerable variation in the width of type V tuning with changes in sound level (green dashed curves in Figure 6C). Weight-function tuning usually broadens near threshold, and in some cases narrows at high sound levels. However, lower frequency edges of weight-function maps remained relatively fixed compared to lower frequency edges of tone-based response maps (*P* < 0.01, signed rank sum test comparisons of slopes of best-fit lines).

Thus, the three CNIC neuron types—I, O, and V—exhibit level-tolerant tuning in response to broadband stimuli, but not in response to tones. For comparison, the edges of weight-function maps for a population of ANF were averaged and plotted in Figure 6B (red). For ANFs, the upper and lower frequency edges delineate an area of excitation that is centered on BF. The ANF weight-function map broadens slightly with increasing noise level at the lower frequency edge, exhibiting more widening than observed in type I and O edges (*P* = 0.02; signed rank sum test of slopes of best-fit lines). By contrast, ANF tuning widens less than that of type V neurons (*P* = 0.07).

### Quality of model predictions and the importance of 2nd-order terms

**Figure 7:**
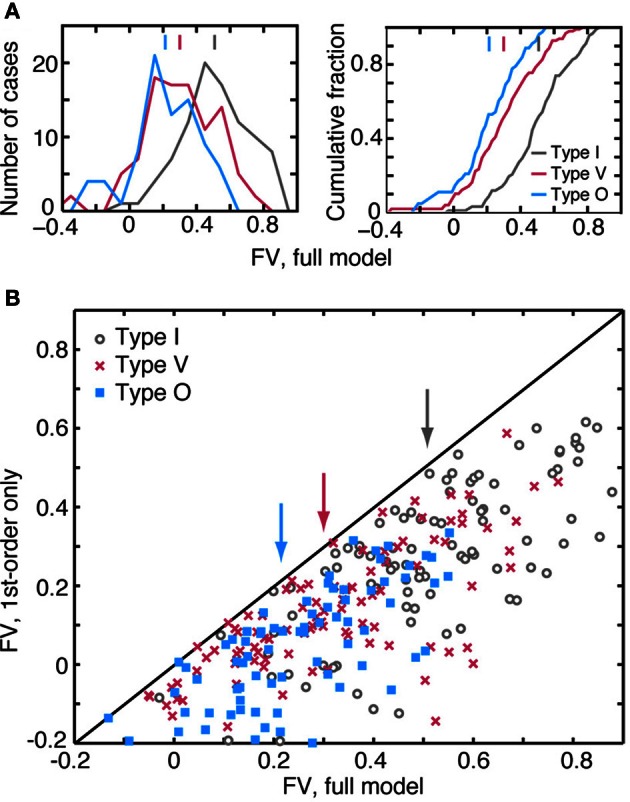
**Quality of the model predictions as measured by *fv* in leave-one-out cross validation tests.** Each plot contains an aggregation of prediction data obtained at different sound levels. A neuron is usually represented multiple times, once at each sound level and also at pairs of adjacent sound levels. **(A)** Distribution (left) and cumulative distribution (right) of *fv*-values for the three response types. Vertical lines at the top of the plots show median *fv*-values. Differences in the distributions across the neuron types are statistically significant (rank sum test with Bonferroni correction; I vs. V and I vs. O, *P* << 0.001; V vs. O, *P* < 0.02). **(B)** Comparison of the best prediction quality (*fv*) for a full binaural model containing all 2nd-order terms (abscissa) and a model containing only 1st-order terms (ordinate). Ten data points with ordinate values <−0.2 are not shown, but are included in the statistics. Vertical arrows indicate median *fv*-values for each of the neuron types.

The weight-function model in Equation 1 predicts responses of the three CNIC neuron types with different degrees of accuracy. Figure 7A shows the distributions (left) and cumulative distributions (right) of *fv*-values for predictions computed using the leave-one-out procedure. Of the three response types, the model performs best for type I neurons where the median value of *fv* is 0.51 (gray). For the type V and O populations, median values of *fv* are only 0.30 (red) and 0.21 (blue), respectively. The differences in the medians of the *fv* distributions are statistically significant (see the figure caption).

The accuracy of the model predictions depends substantially on the inclusion of 2nd-order terms. The scatterplot of Figure 7B compares the performance of two models for each test response—a model which includes all 1st- and 2nd-order terms (abscissa), and another which includes only the 1st-order terms (ordinate). All data points are located below the diagonal line, meaning that the addition of 2nd-order terms consistently improves model performance. This was found to be true regardless of the quality of the 1st-order fit or the neuron type. Specifically, the median improvement in *fv* (calculated as the difference in *fv*-values for the 2nd- and 1st-order models) is 0.23 for I, 0.16 for V, and 0.18 for O neurons. Although it is not shown here, the binaural term (i. e., the sixth term of Equation 1) has a negligible impact on the quality of the prediction. In 225/310 cases, the improvement in *fv* resulting from the addition of binaural weights was less than 0.01.

**Figure 8:**
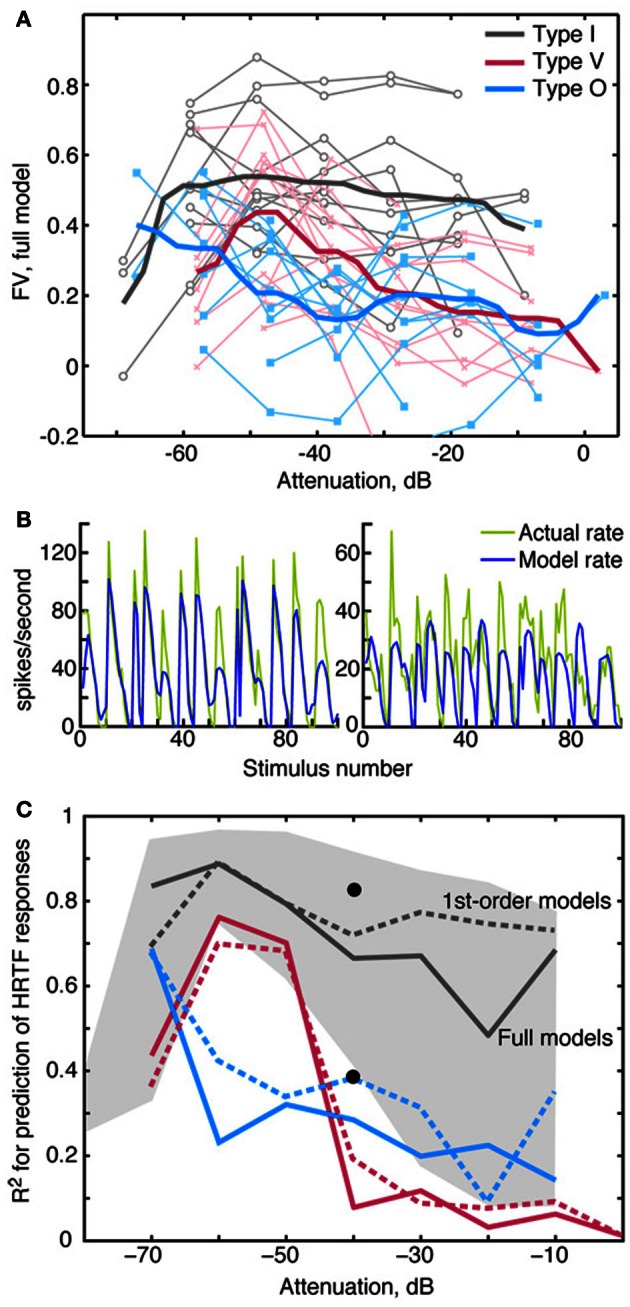
**Prediction quality for RSS and HRTF stimuli by sound level. (A)** Quality of RSS response predictions (*fv*) plotted against the sound level of the stimulus. Light colored symbols connected by lines show data obtained from one neuron. Heavy colored lines show median *fv*-values for the 3 neuron types. For clarity in plotting, 1 dB was added to the attenuations for V neurons, and 2 dB was added to the attenuations for O neurons. **(B)** Predicted rate responses to HRTF-filtered noise (VS stimuli), computed using weight-function models derived at the same overall sound level (−40 dB). Predictions for a type I (left) and a type O (right) neuron were computed using the difference method (see text). Actual rates are shown in green and predicted rates are shown in blue. The goodness of fit for these predictions are as follows: Type I (left): *R*^2^ = 0.82, *fv* = 0.7; type O (right): *R*^2^ = 0.39, *fv* = 0. **(C)** Median *R*^2^ for the three neuron types. Solid lines indicate median *R*^2^-values obtained when the full model was used to predict responses to VS stimuli; dashed lines indicate median *R*^2^-values based only on 1st-order terms. The black dots show the positions of the two examples in **(B)**. The gray shaded region indicates the range of *R*^2^-values that were observed for type I neurons at each attenuation level.

Generally the accuracy of model predictions declines as sound level increases. Figure 8A shows the relationship between prediction accuracy (*fv*) and RSS stimulus level. The weight-function model most accurately predicts responses of type I neurons (gray lines), and this prediction performance declines slightly with sound level (*R* = −0.17, NS). Prediction performance for type V and type O neurons is relatively less accurate across sound levels (red and blue lines), suggesting that nonlinearities not captured by the weight-function model have a marked impact on the rate responses in these neurons. The decline in prediction accuracy with sound level is significant in V neurons (*R* = −0.5, *P* < 0.001), but not in O neurons (*R* = −0.04, NS).

An important test of a model is whether it can predict responses to stimulus types different from those used to estimate model parameters. For this purpose, we tested the model with a functionally relevant stimulus set—specifically, broadband noise filtered by HRTFs (Figure 1D) that simulate natural sound localization cues. As described in Methods, the absence of a reference stimulus for the estimation of *R*_0_ required a modified prediction approach. In Figure 8B, VS response predictions computed in this manner are shown for type I and type O neurons. Whereas rate fluctuations are qualitatively similar between the actual (green lines) and model (blue lines) rates, there are errors in average rate, especially in the type O example (Figure 8B). To deemphasize differences in the means of the actual and predicted rates, the Pearson correlation coefficient *R*^2^—instead of *fv*—was used to quantify the model fit (see Materials and Methods). Figure 8C shows median values of *R*^2^ for the three neurons types. Consistent with Figure 7, the fits are best for type I neurons (gray lines), which maintain good fits (median *R*^2^ > 0.5) over the range of sound levels; however, the range of *R*^2^-values is quite wide, especially at high sound levels (gray shaded region). At each sound level, *R*^2^-values for type V and type O neurons were comparable to those of type I neurons with the poorest fits.

HRTF response predictions, unlike RSS response predictions, were generally not improved with the addition of 2nd-order terms. In fact, at high sound levels, median *R*^2^-values were slightly better for 1st-order models, as shown by the dashed lines in Figure 8C.

### Importance of the contralateral ear

**Figure 9:**
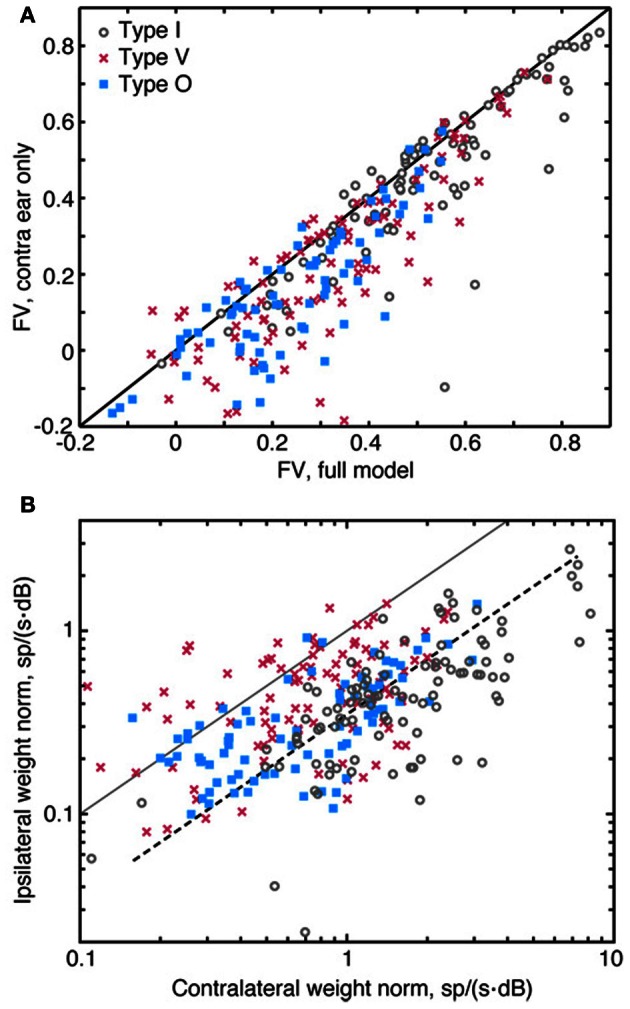
**Relative contribution of ipsilateral and contralateral weights. (A)** Comparison of the prediction quality (*fv*) for a full 2nd-order binaural model (abscissa) and a model which assumes a purely contralateral input [i. e., S_*I*_(*f*) = 0 for all *f*, ordinate]. Data from one experiment showing little or no effect of the ipsilateral inputs are included in this plot. **(B)** Plot of the amplitude of the 1st-order weight vector in the contralateral ear (abscissa) vs. the weight amplitude in the ipsilateral ear (ordinate). The amplitudes of the weights are measured as the norm of 1st-order weight vectors (the square root of the sum of the weights squared). The solid diagonal line indicates where weight vector norms are equal in amplitude. The dashed line indicates where ipsilateral norms are one-fourth the size of contralateral norms. Note the use of logarithmic axes in **(B)**. Also note that data points shown in **(A)** and **(B)** were aggregated across all sound levels of stimulus presentation.

Although it is well known that neurons in the CNIC have tuned responses to both contralateral and ipsilateral stimuli, the importance of ipsilateral inputs to spectral selectivity of neurons has not been studied extensively. We investigated this question by fitting a contralateral-only model to the data—that is, a model incorporating only the first, second, and fourth terms of Equation 1. We then used the monaural model to predict responses to test stimuli and compared the quality of those predictions to the quality achieved using the full binaural model (Figure 9A). The exclusion of ipsilateral information from the model was found to reduce the quality of the predictions: most of the data points in Figure 9A lie below and to the right of the diagonal line. However, the impact of the ipsilateral contribution is relatively small, as inclusion of ipsilateral weights in the model improved *fv* by more than 0.2 for only about 10% of the data.

Ipsilateral weights have a relatively weak impact on the accuracy of the model, partly because they are smaller in magnitude than contralateral weights. This difference in magnitude—which is evident in Figures 2B, 3B, 4B—are summarized in Figure 9B. Here, amplitudes of 1st-order contralateral weight vectors (abscissa) are plotted against amplitudes of 1st-order ipsilateral weight vectors (ordinate). These amplitudes are computed as the norm (or length) of the corresponding weight vector. Only type V neurons frequently exhibit ipsilateral norms that are larger than contralateral norms. By contrast, the ipsilateral norms for type I and type O neurons are usually relatively small. In Figure 9B, the dashed line indicates where ipsilateral norms are one-fourth the size of contralateral norms. For type I and type O neurons, median contralateral and ipsilateral norms (for contralateral norms greater than 0.6) are roughly approximated by this line. The norms of 2nd-order weights behave similarly (not shown).

### Effect of sound level on spectral representations

**Figure 10:**
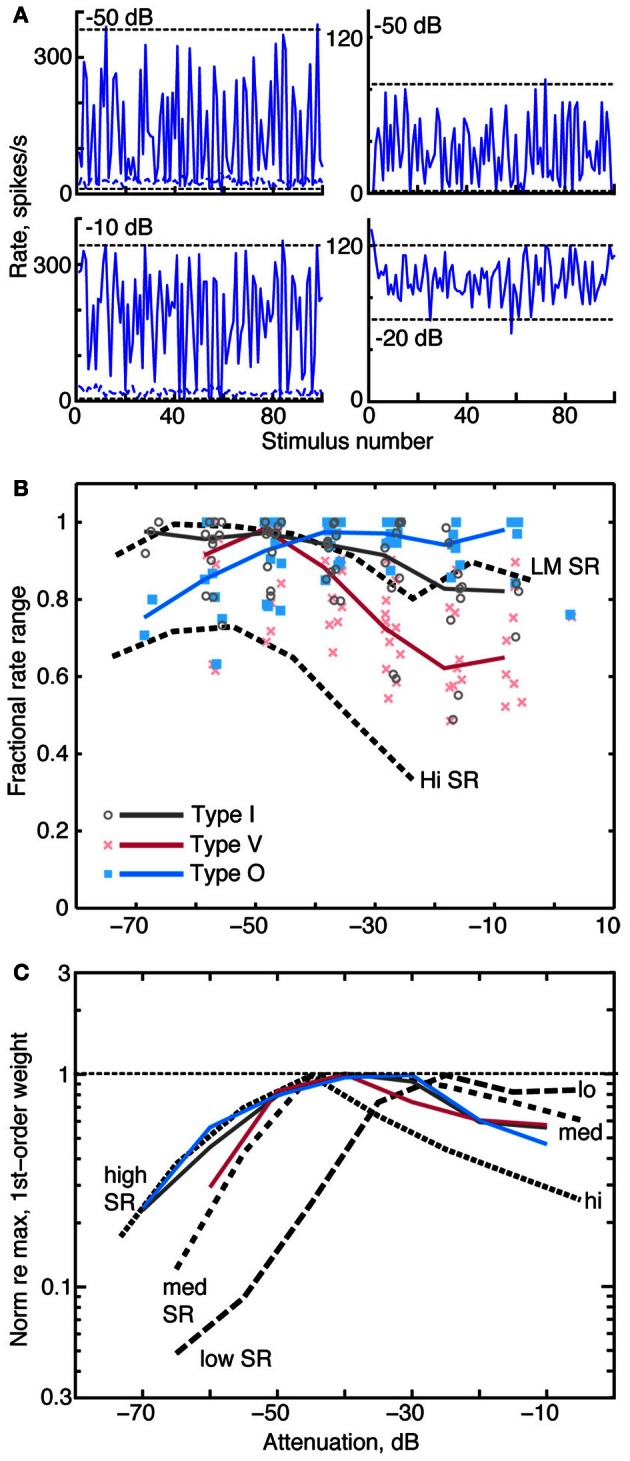
**Relationships between rate dynamic range, weight amplitude, and stimulus level. (A)** Rate responses of two type I neurons to RSS stimuli. Each column contains data from one neuron, presented at two sound levels, as given by the legends. Horizontal dashed lines indicate rates at the 2.5 and 97.5 percentiles of the distribution of rates. *FRR* is calculated from these rates, see text. **(B)** *FRR*s of type I, O, and V populations at different attenuations of the RSS stimuli. Symbols mark the *FRR* of one neuron's responses at the indicated sound level. Abscissa positions of the data points are dithered by ±3 dB to improve clarity. Solid colored lines are the median *FRRs* for the neuron types in 10 dB bins. Black dashed lines are the median *FRR*s of 355 ANFs with high spontaneous rate (*Hi SR*) and low-to-medium spontaneous rate (*LM SR*) [from Young and Calhoun (2005)]. Low and medium SR fibers have been combined because they exhibit identical behavior. **(C)** Median norms of 1st-order weight vectors for CNIC neurons (contralateral only; colored lines) and ANFs [dashed lines; from Young and Calhoun (2005)]. For each neuron type, median norms have been scaled to a maximum value of 1 for comparison. Median norms peak at the following values, in spikes/s/dB: type I, 2.4; type V, 1.4; type O, 1.4; low-SR ANF, 3.0; medium-SR ANF, 3.0; and high-SR ANF, 2.9.

A neuron that provides a robust rate-based spectral representation should respond over as wide a range of discharge rates as possible in response to changing spectral features. The neuron should also adapt the range of response rates to changing stimulus level (Rees and Palmer, 1988; Dean et al., 2008). Each column of Figure 10A shows rate responses of a type I neuron to the RSS stimulus set at two sound levels. The neuron in the left column responds at both sound levels with the full range of rates available to the neuron at the given sound level. By contrast, the neuron in the right column responds at the higher level over roughly half its full rate range due to rate saturation. To evaluate differences in the range of rate responses across sound level, we use the fractional rate ratio (*FRR*), which is defined as ([Rate at 97.5 percentile] – [Rate at 2.5 percentile])/[Rate at 97.5 percentile]. Here, the percentiles (horizontal dashed lines in Figure 10A) are used to reduce the effect of occasional rate outliers. *FRRs* for these examples are 0.97 and 0.98 in the left column and 1.0 and 0.50 in the right column. *FRR* is normalized by the maximum rate at a particular level—not the maximum rate across all levels. The latter would produce an increase in *FRR* at low levels that reflects the strength of the response rather than the neuron's use of the range of rates available to it.

As shown in Figure 10B, the dynamic range of rate responses are well-maintained in CNIC neurons across sound levels. Here, *FRR* is plotted against sound level for individual neurons (symbols), and heavy colored lines indicate the median *FRR*s for each neuron type. Median *FRR*s are also shown for low, medium, and high spontaneous rate ANFs (dashed lines) responding to the same stimulus set.

In type I and type V neurons, the largest *FRRs* occur at low sound levels and decline monotonically at higher levels. The *FRRs*, which tend to be larger for type I than for type V neurons, are generally comparable to those of low and medium spontaneous rate ANF. Type O neurons have the opposite behavior, showing higher *FRRs* at high sound levels. FRR values across the three groups are statistically different at the levels between −30 and 0 dB attenuation (*P* < 0.001 ranksum with Bonferroni correction).

When normalized by their peak value, median contralateral weight vector norms (i. e., amplitudes) of type I, type V, and type O neurons similarly increase at low sound levels and gradually peak between 40 and 30 dB attenuation (Figure 10C; solid colored lines). In a comparison of these amplitudes with those of ANFs (black dashed lines), median amplitudes of type I and O neurons correspond closely with those of high SR fibers at low sound levels. This finding suggests that high SR fibers alone can account for the responses of CNIC neurons to RSS stimuli at low sound levels., However, median amplitudes of high SR fibers decrease rapidly above 50 dB attenuation (presumably as a consequence of rate saturation; Young and Calhoun, 2005) whereas CNIC amplitudes continue to peak. The relationship between weight vector amplitudes and attenuation level at these higher sound levels more closely resembles that of low- and medium-SR fibers. Thus, weight vector amplitudes of CNIC neurons seem to reflect the behavior of the most responsive groups of AN fibers at each sound level.

## Discussion

### The weight-function model

Unlike STRFs, weight-function maps computed in this study do not describe the temporal selectivity of CNIC neurons. Rather, weight functions can be viewed as a frequency marginal of the STRF, providing an average of STRFs across the time dimension (Kim and Young, 1994). In neurons where STRFs are separable—that is, where STRFs can be written as the product of a frequency function, *A(f)*, and time function, *B(t)*, [i. e., *A(f)B(t)*]—weight functions can be viewed as similar to the frequency function *A(f)*. At lower sound levels, STRFs of most CNIC neurons have been found to be separable (Qiu et al., 2003; Lesica and Grothe, 2008a), but as stimulus levels increase, STRFs lose separability (Lesica and Grothe, 2008a). This observation may mirror the complex shape changes observed in RSS-derived weight functions, particularly among type V and O neurons. A clear example of neurons with inseparable characteristics that can't be modeled with weight functions are those with directional responses to frequency sweeps (Andoni and Pollak, 2011). In addition, a strictly spectral model is not useful in describing responses to stimuli with substantial amplitude or frequency modulation (Delgutte et al., 1998; Lesica and Grothe, 2008b; Zheng and Escabi, 2008).

Despite these limitations, the weight-function model has advantages over other approaches to broadband characterization of auditory neurons. One is that it is easy to express the responses in terms of nonlinear stimulus dimensions that are natural for the auditory system, i.e., log frequency and log sound level. The appropriateness of log frequency is clear from the layout of frequencies on the basilar membrane and in central auditory maps. The appropriateness of log sound level is suggested by amplitude compression in the response of the basilar membrane [reviewed by Robles and Ruggero (2001)] and by the fact that discharge rate is locally linear with dB amplitude in AN fibers (Sachs et al., 2006) and neurons in the cochlear nucleus (May et al., 1998). Further evidence is the fact that computing STRFs with a logarithmic stimulus-amplitude scale results in better fits in prediction tests compared to a linear or power scale (Escabi et al., 2003; Gill et al., 2008).

The actual functional form of the best-fitting input nonlinearity was determined from data by Ahrens et al. (2008) in auditory cortex neurons. Their input nonlinearities were expressed as functions on a log stimulus amplitude scale, i.e., as an additional nonlinearity beyond the logarithm. In most cases, the functions were linear over some portion of the log scale, consistent with the model used here, but modified by rectification and sometimes saturation at low and high levels. Another approach to describing input nonlinearities, which was mentioned in the introduction (Bandyopadhyay et al., 2007), is to incorporate an intensity nonlinearity into a 1st-order weight function for neurons in dorsal cochlear nucleus. In this case, the nonlinearity is frequency-dependent and cannot be represented with a single input nonlinearity.

### Importance of second-order filters

Previous studies of CNIC neurons have yielded conflicting perspectives on the importance of 2nd-order filters. In the bat, 2nd-order filters have been shown to be important in describing CNIC neuron responses (Andoni and Pollak, 2011); by contrast, in the cat, 2nd-order filters have been found not to be useful (Atencio et al., 2012). Reasons for these differences are not clear. However, the results of the current study clearly support the view that 2nd-order filters are important in the CNIC. As shown in Figure 7B, the accuracy of the spectral integration model for CNIC neurons is improved with the addition of 2nd-order terms. In fact, in 93% of neurons studied, the addition of one or two judiciously chosen 2nd-order weights increased *fv*-values to levels not achievable through the addition of 1st-order weights alone (data not shown). The latter observation indicates that improvements in prediction accuracy depend specifically on 2nd-order terms and do not merely reflect the inclusion of a larger number of model parameters. Similar improvements to 1st-order models achieved through the inclusion of 2nd-order terms have been described for temporal responses in auditory cortex (Pienkowski et al., 2009).

The improvement in prediction performance achieved with 2nd-order filters may simply reflect the addition of static nonlinearities that match the curvature of a neuron's rate-level function. In models based on the STRF, prediction quality is often improved by following the linear STRF with a static nonlinear function—one that matches the amplitude of the STRF output to the neuron's response rate (e.g., Escabi et al., 2005; Nagel and Doupe, 2006; Lesica and Grothe, 2008a; Sharpee et al., 2008; Atencio et al., 2012). In these so-called “linear-nonlinear models,” the nonlinear segment often bears the shape of a parabola that is dominated by a 2nd-order term. In the current study, the 2nd-order filters are equivalent to such models insofar as the 2nd-order filter and 1st-order weight functions demonstrate the same frequency selectivity. This was sometimes observed in the CNIC, as with the type O neuron in Figure 3D (blue filter, bottom left). However, in almost all cases, 2nd-order filters exhibited frequency selectivity that differed significantly from that of 1st-order weight functions (see part D of Figures 2 through 4). This suggests that a response function in the CNIC often cannot be described by simply appending a single nonlinear filter to a linear one. This finding has important functional implications, as it reveals a complexity in the frequency selectivity of CNIC neurons that is not captured by a 1st-order model alone.

However, the 2nd-order terms in weight functions are not universally useful, as was found for responses to HRTF filtered noise (Figure 8C). Although the addition of 2nd-order terms improved RSS response predictions, this was not true for HRTF response predictions. In fact, on average, weight-function models predicted HRTF responses better without the 2nd-order terms. Errors resulting from the inclusion of 2nd-order terms may reflect inaccuracies in modeling the average rate responses to those stimuli. When 2nd-order terms were added, errors in average rate were found to be larger than gains in the rate fluctuations. This suggests that the estimation of 2nd-order terms is more stimulus-dependent than the estimation of 1st-order terms. This interpretation is consistent with the general finding that STRF models perform better for the stimulus type to which they are fit (e.g., Theunissen et al., 2000; Machens et al., 2004). A similar result was recently obtained while trying to predict the binaural responses of neurons in the nucleus of the brachium of the inferior colliculus (Slee and Young, 2013). Here, the responses to binaural VS were well-predicted by an RSS model with only1st-order terms.

### Binaural interaction

As in previous studies of decerebrate cats (Davis et al., 1999; Chase and Young, 2005), CNIC neurons in the present sample were found to be binaural: that is, monaural stimuli presented to either ear yielded a response with a clear BF. Frequency selectivity in binaural CNIC neurons is often studied using monaural stimuli, where stimuli are presented either to the contralateral ear alone or in free field. But auditory neurons rarely encounter stimuli that are truly monaural in a natural environment. Studies involving binaural free-field stimulation, which are often based on a more natural stimulus presentation, may also be problematic in that they usually do not account for the varying dichotic nature of the stimuli. As such, responses dependent on the interaction of contralateral and ipsilateral inputs—e. g., sensitivity of neurons to interaural level differences (Delgutte et al., 1999)—are often not analyzed in a systematic manner that is frequency-specific.

In this work, independent RSS stimuli were presented to the two ears simultaneously. This binaural stimulus presentation enabled interactions between contralateral and ipsilateral inputs—as well as contralateral and ipsilateral frequency selectivity—to be investigated in a controlled manner. Furthermore, frequency selectivity, which was computed in the form of weights, could be compared between the two ears. For type I and O neurons, contralateral weights were almost always found to be larger in magnitude than ipsilateral weights (Figure 9B), suggesting that the responses of these neurons are more strongly influenced by spectra presented to the contralateral ear. Studies of CNIC neurons based on STRFs corroborate this finding, indicating that ipsilateral STRFs—which were found to be significant for only 36% of CNIC neurons studied—usually provide weak spectral representations (Qiu et al., 2003). For some binaural CNIC neurons, the contralateral weight functions alone appear sufficient to describe rate responses (e. g., the points on the diagonal line in Figure 9A), suggesting that these neurons are weakly responsive to ipsilateral RSS inputs. Prediction testing does suggest, however, that inputs from the ipsilateral ear contribute significantly to the responses of many CNIC neurons. Figure 9A indicates that spectral integration models that ignore inputs to the ipsilateral ear may yield errors in a large number of neurons. Difficulties reported in using STRFs to predict CNIC responses may reflect, to some extent, omission of ipsilateral inputs.

The consistently small size of binaural interaction weights (i. e., *b*_*jk*_, the sixth term in Equation 1) suggests that binaural interactions, as they pertain to stimulus spectra (i. e., interaural level differences), are primarily additive rather than multiplicative. This conclusion is subject to the reservation that binaural interactions related to interaural time differences are not addressed here.

### Level-tolerant tuning

The tuning of response areas—whether derived from noise or tone stimuli—becomes more level tolerant across the ascending pathway from the cochlea to the auditory cortex. For tones, neurons show sharper tuning in CNIC than in the cochlea (measured by Q40; Ramachandran et al., 1999; McLaughlin et al., 2007), and fully level-tolerant tone response maps have only been reported in auditory cortex (Sutter, 2000; Sadagopan and Wang, 2008). Tuning to broadband stimuli is more level tolerant than tuning to tones, even at the level of the auditory nerve (Carney and Yin, 1988; Young and Calhoun, 2005) where level tolerance is believed to reflect cochlear suppression. In this study, we show weight-function maps that are more level tolerant than auditory nerve maps (Figure 6B), and tuning in type I and O neurons that is fully level tolerant. Experiments based on antagonists to inhibitory neurotransmitters (Yang et al., 1992; Lebeau et al., 2001), small cochlear lesions (Snyder and Sinex, 2002), and stimulus-driven adaptation suggest that sharpening of tone tuning can be attributed to inhibitory inputs at frequencies away from BF. The level tolerance observed in CNIC weighting functions is likely also shaped by this inhibition. From a functional perspective, level tolerance is significant: it suggests that CNIC neurons are capable of maintaining selectivity for narrow spectral features—such as those found in HRTFs—across a wide range of stimulus levels.

### Response types in the CNIC

The results shown here add to the current understanding of neural response types in the cat CNIC. The classification scheme applied in this study is based on spectral tuning for tones (Ramachandran et al., 1999; Greene et al., 2010), analysis of masking patterns (Ramachandran et al., 2000), and binaural processing (Davis et al., 1999; Ramachandran and May, 2002). The validity of this scheme is supported by the fact that class definitions incorporate multiple aspects of a neuron's response properties. Moreover, similar response types have been described in a number of animal species (when effects of anesthesia and stimulus design are taken into account; for example: bat, Yang et al., 1992; guinea pig, Lebeau et al., 2001; mouse, Egorova et al., 2001; rat, Hernández et al., 2005). The relative prevalence of the three response types does varies widely across species (Davis, 2005). Neurons resembling types I and V are found in all species, but the prevalence of type O neurons, which are most common in cats, varies significantly. This suggests that there are significant species differences in the representation of sound in IC.

In contrast to tone maps, the shapes of weight-function maps as described in this study do not clearly define the response types nor allow them to be differentiated. In type I neurons, weight-function maps usually exhibit on-BF positive (excitatory) peaks for the contralateral ear and on-BF negative troughs (inhibition) for the ipsilateral ear (as in Figure 2). It can also be stated that in type O and type V neurons, contralateral and ipsilateral weight-function maps usually contain areas of inhibition near BF that are more prominent than those for type I neurons (as in Figures 3, 4). However, patterns of excitation and inhibition observed in weight-function maps—within a single response type—vary significantly, particularly within the type V and O classes. As a result, weight-function maps do not in themselves support a well-defined classification scheme. Likewise, studies of STRFs have yet to define a classification scheme for auditory neurons in CNIC or elsewhere [although see Woolley et al. (2009) in bird cortex].

On the other hand, results of this study do support the functional relevance of the type I, V, and O classification scheme. Specifically, the nature of spectral sensitivity differs for the three classes. As demonstrated in Figures 7, 8, each of the neuron types is sensitive to spectral shape, as rate responses vary with changes in stimulus spectrum. For type I neurons, a low-order model containing only 1st- and 2nd-order terms typically accounts for most of the variance in rate responses—and thus spectral sensitivity—at stimulus levels up to 50–60 dB above threshold. By comparison, a low-order model is a less accurate description of type V and O rate responses, except at the lowest stimulus levels (Figure 8). The nature of spectral encoding therefore appears to be different for type I, V, and O neurons.

Differences in spectral encoding between type I, V, and O neurons support the idea of parallel representations of auditory stimuli (Yu and Young, 2000; Escabi and Schreiner, 2002; Woolley et al., 2009). Type I neurons, like chopper neurons in the ventral cochlear nucleus, produce linear representations of spectral shape. The weight function model did successfully capture the variation in rate responses across a set of untrained, functionally relevant VS stimuli (see Figures 8B,C). On the other hand, type O and V neurons exhibit nonlinear relationships between spectral level and discharge rate that imply other functional possibilities. One possibility is that “nonlinear” neurons may be encoding specific features of the stimulus—e. g., spectral notches or rising spectral edges, as are present in VS stimuli—in a manner not easily explained with a linear spectral integration model. Such neurons have been observed in the dorsal cochlear nucleus and CNIC (Davis et al., 2003; Escabi et al., 2005; Reiss and Young, 2005). Alternatively, the responses of nonlinear neurons may be more strongly influenced by properties of the stimulus other than the spectra, such as features encoded in the time domain. For example, type V neurons seem to be specialized for encoding interaural time differences (Ramachandran and May, 2002; Chase and Young, 2005), which were not controlled in RSS stimuli presentations. A full exploration of the link between response nonlinearity, feature selectivity, and temporal sensitivity remains to be done.

### Conflict of interest statement

The authors declare that the research was conducted in the absence of any commercial or financial relationships that could be construed as a potential conflict of interest.
